# Identification of clinical and radiographic predictors of central nervous system injury in genetic skeletal disorders

**DOI:** 10.1038/s41598-021-87058-5

**Published:** 2021-05-31

**Authors:** Antônio L Cunha, Ana P S Champs, Carla M. Mello, Mônica M. M. Navarro, Frederico J. C. Godinho, Cássia M. B. Carvalho, Teresa C. A. Ferrari

**Affiliations:** 1 Rede SARAH de Hospitais de Reabilitação, Av. Amazonas, 5953. Gameleira, Belo Horizonte, MG 30510-000 Brazil; 2 Centro de Pós-Graduação, Faculdade de Medicina, Universidade Federal de Minas Gerais, Av. Prof. Alfredo Balena, 190. Santa Efigênia, Belo Horizonte, MG 30130-100 Brazil

**Keywords:** Genetics, Neurology

## Abstract

Some studies report neurological lesions in patients with genetic skeletal disorders (GSDs). However, none of them describe the frequency of neurological lesions in a large sample of patients or investigate the associations between clinical and/or radiological central nervous system (CNS) injury and clinical, anthropometric and imaging parameters. The project was approved by the institution’s ethics committee (CAAE 49433215.5.0000.0022). In this cross-sectional observational analysis study, 272 patients aged four or more years with clinically and radiologically confirmed GSDs were prospectively included. Genetic testing confirmed the diagnosis in the *FGFR3* chondrodysplasias group. All patients underwent blinded and independent clinical, anthropometric and neuroaxis imaging evaluations. Information on the presence of headache, neuropsychomotor development (NPMD), low back pain, joint deformity, ligament laxity and lower limb discrepancy was collected. Imaging abnormalities of the axial skeleton and CNS were investigated by whole spine digital radiography, craniocervical junction CT and brain and spine MRI. The diagnostic criteria for CNS injury were abnormal clinical and/or radiographic examination of the CNS. Brain injury included malacia, encephalopathies and malformation. Spinal cord injury included malacia, hydrosyringomyelia and spinal cord injury without radiographic abnormalities. CNS injury was diagnosed in more than 25% of GSD patients. Spinal cord injury was found in 21.7% of patients, and brain injury was found in 5.9%. The presence of low back pain, os odontoideum and abnormal NPMD remained independently associated with CNS injury in the multivariable analysis. Early identification of these abnormalities may have some role in preventing compressive CNS injury, which is a priority in GSD patients.

## Introduction

Genetic skeletal diseases (GSDs) include dysostoses (focal bone abnormalities of one or a group of bones), skeletal dysplasias (abnormalities that affect the development of bones and cartilage tissues), disruptions (malformations of bones secondary to nonskeletal causes) and osteolyses (regressive and resorptive bone abnormalities)^1^. Patients with GSDs are at an increased risk of brain, spinal cord and spinal root injury^2–4^. These neurological injuries can occur even in the absence of trauma^5^. The interpretation of skull, craniocervical junction (CCJ) and spine radiological abnormalities is challenging and, at the same time, essential to planning monitoring and treatment strategies^6–9^.

In individuals with GSDs, the primary mechanisms of nervous system injury^2,5,6,10–13^ are driven by gene mutations. There are also secondary mechanisms related to skeletal abnormalities^2–5,10^. Neurological injury may occur, especially when the following characteristics are present: bone fragility, which increases the risk of skull and vertebrae fractures^3,14,15^; ligament laxity, which is associated with instability and dislocations of the CCJ and between vertebrae^3,4,16–19^; an abnormal configuration of the skull and vertebrae, such as that in cases of platybasia, basilar impression/invagination (BII), an abnormal odontoid process and platyspondyly^2,7,10,15,17,18,20–25^; abnormal spinal curvatures, which change the distribution of the axial load and compromise the spinal balance^2,3,10,26–29^; and disorganized development of the skeletal components, such as that in cases of osteochondromas, which grow inside the skull and vertebrae^30–40^. It is also necessary to be aware that the findings of neurological examinations can be affected by cognitive, consciousness and/or osteoarticular impairments^41,42^ and that there are patients with clinical evidence of spinal cord injury without radiographic abnormalities (SCIWORA)^4,6,43^. The most common brain abnormalities that can be detected by imaging in GSD^10^ are the following: enlargement of the ventricular system (ventriculomegaly and hydrocephalus), structural brain abnormalities (holoprosencephaly, agenesis and hypoplasia of the cerebellum and brain components, malformations of cortical development, abnormalities of neuronal migration, cerebellar tonsillar ectopia and arachnoid cysts), cortical and brain white matter signal intensity abnormalities (demyelination and hypomyelination), ischemic lesions (gliosis, encephaloma and hypoxia) and cerebrovascular abnormalities.

Radiologists should be included in the multidisciplinary team that takes care of these patients. Imaging methods allow the description of abnormalities of the axial skeleton and of the central nervous system (CNS)^10,44–47^. The implications of imaging findings can be better interpreted in the context of clinical abnormalities detected by neurological examination^4–6,43,48^. Strategies to ensure that GSD patients have a longer and better-quality life are largely based on early identification of CNS compression before the establishment of damage to the nerve tissue.

Thus, GSD patients constitute a diverse population at high risk for neurological injury and are treated by health care providers with different medical specialties to increase patients’ life expectancy^49^. Considering the early age at which neurological damage may occur, the severity of the sequelae, and the high cost of rehabilitation and orthopedic and urological treatment, it is important to identify skeletal axis changes that can cause CNS injury.

The aims of this study were to describe the neurological lesions diagnosed clinically and/or by imaging methods in a large sample of GSD patients and to investigate the associations between CNS injury and clinical manifestations, anthropometric measures and imaging findings.

## Results

A total of 272 patients, 146 (54%) of whom were female, in the 25 GSD groups^50^, as listed in Table 1, were included in the study. The ages of the patients ranged from five to 70 years, with an asymmetric distribution across decades (a larger number of patients in the second and third decades). The mean age of the patients was 25 years, with a standard deviation (SD) of 13.6 years, and the median age was 22 years (interquartile range [IQR] 15–32 years). There were 28 (10.3%) patients under 10 years of age and 53 (19.5%) between 10 and 15 years old.

**Table 1:** Genetic skeletal disorder groups and abnormal neuropsychomotor development frequency.

Number/genetic skeletal disorder groups^a^	n	%	ANPMD	%
26. Abnormal mineralization group	67	24.6	6	9.0
29. Disorganized development of skeletal components group	44	16.2	2	4.5
25. Osteogenesis imperfecta and decreased bone density group	38	14.0	5	13.2
13. Spondylo-epi-(meta)-physeal dysplasias	23	8.5	–	–
10. Multiple epiphyseal dysplasia and pseudoachondroplasia group	19	6.9	1	5.3
1. *FGFR3* chondrodysplasias group	20	7.4	3	15.0
11. Metaphyseal dysplasias	11	4.0	1	9.1
12. Spondylometaphyseal dysplasias	11	4.0	–	–
27. Lysosomal storage diseases with skeletal involvement (dysostosis multiplex)	9	3.3	6	66.7
5. Perlecan group	5	1.8	1	20.0
23. Osteopetrosis and related disorders	5	1.8	–	–
15. Acromelic dysplasias	4	1.5	–	–
4. Sulfation disorder group	3	1.1	–	–
32. Cleidocranial dysplasia and related disorders	3	1.1	–	–
8. *TRPV4* group	2	0.7	1	50.0
9. Ciliopathies with major skeletal involvement	1	0.4	1	100.0
24. Other sclerosing bone disorders	1	0.4	1	100.0
2. Type 2 collagen group and 3. Type 11 collagen group	1	0.4	1	100.0
16. Acromesomelic dysplasias	1	0.4	–	–
17. Mesomelic and rhizo-mesomelic dysplasias	1	0.4	–	–
28. Osteolysis group	1	0.4	–	–
37. Brachydactylies (without extraskeletal manifestations)	1	0.4	1	100.0
42. Defects in joint formation and synostosis	1	0.4	–	–

*ANPMD* abnormal neuropsychomotor development, *FGFR3* fibroblast growth factor receptor 3, *TRPV4* transient receptor potential action channel subfamily V member 4.

^a^According to the 2019 version of the Classification and Nosology of the ISSD^50^.

CNS injury was detected in 71 (26.1%) patients, as shown in Table 2. Spinal cord injury in 59 (21.7%) patients occurred in a larger percentage of patients than brain injury (16 patients, 5.9%). Brain and spinal cord injuries were simultaneously detected in four (1.5%) patients. Cervical SCI was more frequent in 13 (4.8%) patients than thoracic SCI in one (0.4%) patient. Thoracic hydrosyringomyelia was more frequent in 10 (3.7%) patients than cervical hydrosyringomyelia in four (1.5%) patients. Spine MRI showed both cervical and thoracic hydrosyringomyelia in six (2.2%) individuals. Myelomalacia and hydrosyringomyelia were simultaneously detected in one (0.4%) patient. SCIWORA was found in 26 (9.6%) patients.

**Table 2:** The frequency of CNS injury in genetic skeletal disorder patients.

Genetic skeletal disorder patients	n	%
Central nervous system injury	71	26.1
**Spinal cord injury (myelomalacia + hydrosyringomyelia + SCIWORA)**	59	21.7
Myelomalacia (cervical + thoracic)	14	5.2
Cervical myelomalacia	13	4.8
Thoracic myelomalacia	1	0.4
Hydrosyringomyelia (cervical + thoracic + cervical and thoracic)	20	7.4
Thoracic hydrosyringomyelia	10	3.7
Cervical hydrosyringomyelia	4	1.5
Cervical and thoracic hydrosyringomyelia	6	2.2
Spinal cord injury without radiographic abnormalities (SCIWORA)	26	9.6
**Brain injury**	16	5.9
Encephalomalacia	8	2.9
Diffuse white matter abnormalities	3	1.1
Malformation	5	1.8

Brain and spinal cord injury simultaneously occurred in four cases (1.5%), and myelomalacia and hydrosyringomyelia occurred in one case (0.4%).

*CNS* central nervous system.

Encephalomalacia was observed in eight (2.9%) and diffuse white matter alterations in three (1.1%) patients among the brain MRI with detection of injuries. The brain malformations found were imaging findings in five (1.8%) patients, including Chiari 1 in one participant, disorders of forebrain induction (holoprosencephaly) in one patient and cortical development malformation in three patients, none of which was related to a specific GSD. There were no hypoxic ischemic or perinatal brain injuries. There were arachnoid cysts in 10 (3.7%) patients and ventriculomegaly in 23 (8.5%) patients. Cerebral sulcus enlargement was observed in 21 (7.7%) patients, and subarachnoid space widening was observed in 30 (11.0%) patients.

Shorter stature and smaller wingspan occurred more frequently in the patients with CNS injury than in those without CNS injury, as described in Table 3.

**Table 3:** Comparison of anthropometric measures between genetic skeletal disorder patients with and without CNS injury.

Anthropometry	CNS injury Md (IQR)	No CNS injury Md (IQR)	p*
Stature	144 (131–156)	146 (131–158)	0.043
Wingspan	147 (131–161)	149 (132–162)	0.047
Lower segment length	77 (68–84)	78 (69–86)	0.051
Body mass index	24 (20–28)	24 (20–27)	0.081
OFC	56 (54–57)	55 (54–57)	0.085
Upper segment length	71 (65–76)	72 (65–77)	0.089
Trunk length	47 (42–51)	47 (42–51)	0.483
Weight	49 (39–63)	50 (40–63)	0.545

*CNS* central nervous system, *Md* median, *Q1* first quartile, *Q3* third quartile, *OFC* occipitofrontal circumference.

*Mann–Whitney–Wilcoxon test.

Low back pain (OR = 2.3; 95% CI = 1.3–4.2) and abnormal neuropsychomotor development (ANPMD) (OR = 2.8; 95% CI = 1.2–6.1) were associated with a high chance of CNS injury being present, as shown in Table 4.

**Table 4:** Comparison of clinical manifestations between genetic skeletal disorder patients with and without CNS injury.

Clinical manifestations	CNS injury n (%)	No CNS injury n (%)	p*	OR (95% CI)
Low back pain	40/71 (56.3)	72/201 (35.8)	0.003	2.3 (1.3–4.2)
ANPMD	14/71 (19.7)	16/201 (8.0)	0.007	2.8 (1.2–6.1)
Lower limb discrepancy	37/71 (52.1)	80/201 (39.8)	0.072	2.8 (1.2–6.6)
Ligament laxity	14/71 (19.7)	30/201 (14.9)	0.345	1.4 (0.6–3.0)
Joint dislocation	6/71 (8.5)	11/201 (5.5)	0.396	1.6 (0.5–4.9)
Joint deformity	56/71 (78.9)	149/201 (74.1)	0.425	1.3 (0.7–2.7)
Headache	23/71 (32.4)	69/201 (34.3)	0.767	0.9 (0.5–1.7)

*CNS* central nervous system, *ANPMD* abnormal neuropsychomotor development, *OR* odds ratio, *CI* confidence interval.

*X^2^ test or Fisher’s exact test.

There was ANPMD in 30 (11%) patients in the 13 GSD groups. The absolute and relative incidences of ANPMD were higher among the patients with lysosomal storage diseases with skeletal abnormalities (66.7%), perlecan protein abnormalities (20%), *FGFR3* chondrodysplasias (15%) and osteogenesis imperfecta/decreased bone density (13.2%), as shown in Table 1. Clinical manifestations that may be due to neurological injury, such as muscle weakness, the need for mobility aids, difficulty walking, and changes in sensitivity, were not included in the analysis because these manifestations were used for the diagnosis of CNS injury.

Radiological variables that were associated with an increased probability of CNS injury were the presence of spinal abnormalities (OR = 2.1; 95% CI = 1.2–3.9) and abnormal epiphyses (OR = 1.3; 95% CI = 0.3–1.8), as described in Table 5.

**Table 5:** Comparison of radiological abnormalities between genetic skeletal disorder patients with and without CNS injury.

Radiological abnormalities	CNS injury n (%)	No CNS injury n (%)	p*	OR (95% CI)
Spine	41/71 (57.7)	78/201 (38.8)	0.006	2.1 (1.2–3.9)
Epiphyseal	23/71 (32.4)	40/201 (19.9)	0.032	1.3 (0.3–1.8)
Metaphyseal	34/71 (47.9)	124/201 (61.7)	0.043	0.6 (0.3–1.0)
Skull	14/71 (19.7)	22/201 (10.9)	0.061	2.0 (0.9–4.4)
Decreased bone density	21/71 (29.6)	82/201 (40.8)	0.125	0.6 (0.3–1.1)
Diaphyseal	13/71 (18.3)	28/201 (13.9)	0.440	1.4 (0.6–3.0)
Disorganized bone development	10/71 (14.1)	36/201 (17.9)	0.460	0.8 (0.3–1.7)
Increased bone density	2/71 (2.8)	9/201 (4.5)	0.734	0.6 (0.1–3.1)

*CNS* central nervous system, *OR* odds ratio, *CI* confidence interval.

*X^2^ test or Fisher’s exact test.

In the CCJ, as shown in Table 6, the presence of os odontoideum (OR = 4.2; 95% CI = 1.4–11.1), BII (OR = 2.4; 95% CI = 1.1–5.3) and a narrowed FM (OR = 2.2; 95% CI = 1.0–5.0) was associated with a higher probability of CNS injury. Cervical neurological compression occurred in 21.3% of the patients in both groups, with (23.9%) and without (20.4%) CNS injury.

**Table 6:** Comparison of the craniocervical junction abnormalities between genetic skeletal disorder patients with and without CNS injury.

Craniocervical junction abnormalities	CNS injury n (%)	No CNS injury n (%)	p*	OR (95% CI)
Os odontoideum	11/71 (15.5)	9/201 (4.5)	0.006	4.2 (1.4–11.1)
Basilar impression/invagination	15/71 (21.1)	20/201 (10.0)	0.016	2.4 (1.1–5.3)
Narrowed foramen magnum	14/71 (19.7)	20/201 (10.0)	0.032	2.2 (1.0–5.0)
Spinal canal stenosis at C2	59/71 (83.1)	155/201 (77.1)	0.290	1.5 (0.7–3.2)
Atlanto-odontoid instability	6/71 (8.5)	10/201 (5.0)	0.377	1.8 (0.5–5.6)
Cervical spinal cord compression	17/71 (23.9)	41/201 (20.4)	0.531	1.2 (0.6–2.4)
Atlanto-occipital instability	3/71 (4.2)	6/201 (3.0)	0.701	1.4 (0.2–6.9)
Basio-occipital hypoplasia	41/71 (57.7)	127/201 (60.7)	0.768	0.9 (0.5–1.6)
Terminal bone at C2	2/71 (2.8)	4/201 (2.0)	1.000	0.9 (0.1–5.4)
Platybasia	1/71 (1.4)	4/201 (2.0)	1.000	0.7 (0.1–7.3)

*CNS* central nervous system, *OR* odds ratio, *CI* confidence interval.

*X^2^ test or Fisher’s exact test.

Platyspondyly (OR = 2.3; 95% CI = 1.1–4.7) and thoracic osteochondrosis (OR = 2.0; 95% CI = 1.1–3.6) in the spinal imaging findings were associated with a higher chance of CNS injury, as shown in Table 7. Bony spinal canal stenosis was found in 40.4% of the participants and was more frequent in the group with CNS injury (43.7%) than in the group without neurological lesions (39.3%). Discogenic spinal canal stenosis was found in 58.5% of the MRIs performed in the sample and was more frequent in the group with CNS injury (64.8%) than in the group without this lesion (56.2%). Discogenic cervical spinal canal stenosis was observed in 50.7%, thoracic stenosis in 39.4% and lumbar stenosis in 33.8% of the group with CNS injury. Ligamentous spinal canal stenosis was described in 18.0% of the participants and was more frequent in the group with CNS injury (19.7%) than in the group without injury (17.0%). Spinal vertebral compression occurred in 50 (18.4%) GSD patients but was not associated with CNS injury (Table 7).

**Table 7:** Comparison of radiological spinal abnormalities between genetic skeletal disorder patients with and without CNS injury.

Radiological spinal abnormalities	CNS injury n (%)	No CNS injury n (%)	p*	OR (95% CI)
Platyspondyly	18/71 (25.4)	26/201 (12.9)	0.015	2.3 (1.1–4.7)
Cervical platyspondyly	16/71 (22.5)	19/201 (9.5)	0.005	2.8 (1.2–6.1)
Thoracic platyspondyly	18/71 (25.4)	23/201 (11.4)	0.005	2.6 (1.2–5.5)
Lumbar platyspondyly	18/71 (25.4)	21/201 (10.4)	0.002	2.9 (1.3–6.2)
Thoracolumbar kiphosis	15/71 (21.1)	25/201 (12.4)	0.076	1.9 (0.9–4.0)
Scoliosis	36/71 (50.7)	122/201 (60.7)	0.142	0.7 (0.4–1.2)
Osteochondrosis intervertebral	46/71 (64.8)	113/201 (56.2)	0.208	1.4 (0.8–2.6)
Cervical osteochondrosis	36/71 (50.7)	82/201 (40.8)	0.148	1.5 (0.8–2.7)
Thoracic osteochondrosis	28/71 (39.4)	50/201 (24.9)	0.020	2.0 (1.1–3.6)
Lumbar osteochondrosis	24/71 (33.8)	57/201 (28.4)	0.389	1.3 (0.7–2.4)
Spinal vertebrae compression	11/71 (15.5)	39/201 (19.4)	0.465	0.8 (0.3–1.6)
Cervical compression	0/71 (0.0)	4/201 (2.0)	0.558	0.0 (0.0–4.3)
Thoracic compression	10/71 (14.1)	35/201 (17.4)	0.516	0.8 (0.3–1.7)
Lumbar compression	5/71 (7.0)	17/201 (8.5)	0.805	0.8 (0.2–2.4)
Spinal canal stenosis	31/71 (43.7)	79/201 (39.3)	0.520	1.2 (0.7–2.1)
Cervical stenosis	24/71 (33.8)	57/201 (28.4)	0.389	1.3 (0.7–2.4)
Thoracic stenosis	17/71 (23.9)	37/201 (18.4)	0.315	1.4 (0.7–2.8)
Lumbar stenosis	12/71 (16.9)	35/201 (17.4)	0.922	1.0 (0.4–2.1)

*CNS* central nervous system, *OR* odds ratio, *CI* confidence interval.

*X^2^ test or Fisher’s exact test.

The results of the uni- and multivariable analyses are presented in Table 8, and additional data are provided in supplementary materials (Supplementary Tables S1 and S2). The variables that were included in the multivariable model were low back pain, the presence of os odontoideum, ANPMD, radiological spine abnormalities, platyspondyly, BII, epiphyseal abnormalities, narrowed FM, and thoracic osteochondrosis. This model explained only 10.9% of the variance in the outcome (CNS injury). The presence of low back pain (OR = 2.8; 95% CI = 1.3–3.3), ANPMD (OR = 3.0; 95% CI = 1.2–7.9) and os odontoideum (OR = 2.8; 95% CI = 1.3–6.3) was independently associated with the presence of CNS injury.

**Table 8:** Predictors of CNS injury in genetic skeletal disorder patients.

Predictors	Univariable analysis OR (95% CI)	p*	Multivariable analysis OR (95% CI)	p*
Low back pain	2.3 (1.3–4.0)	0.003	2.8 (1.3–3.3)	0.001
Os odontoideum	3.9 (1.5–9.8)	0.004	3.0 (1.2–7.9)	0.021
ANPMD	2.8 (1.3–6.2)	0.008	2.8 (1.3–6.3)	0.012
Radiological spine abnormalities	2.2 (1.2–3.7)	0.006	–	–
Platyspondyly	2.3 (1.2–4.5)	0.016	–	–
Basilar impression/invagination	2.4 (1.2–5.0)	0.018	–	–
Epiphyseal abnormalities	1.9 (1.1–3.5)	0.034	–	–
Narrowed foramen magnum	2.2 (1.1–4.7)	0.036	–	–
Thoracic osteochondrosis	2.0 (1.1–3.5)	0.021	–	–

R^2^ = 10.9%; Hosmer–Lemeshow test = 0.995.

*CNS* central nervous system, *OR* odds ratio, *CI* confidence interval, *ANPMD* abnormal neuropsychomotor development.

*X^2^ test or Fisher’s exact test.

## Discussion

Clinical and/or radiological parameters associated with neurological injury were assessed in GSD patients. A large population who attended a referral rehabilitation center underwent clinical and neuroaxis imaging examinations on the same day, and the data were analyzed systematically using blinding methods; thus, it was possible to describe the occurrence of CNS injury; primary and secondary brain and spinal cord injuries; spinal nerve root compression caused by spinal canal stenosis; and SCIWORA.

More than 25% of GSD patients had CNS injury. Early identification of these anomalies is essential to prevent CNS injury caused by compression of the CNS before the neurological lesion is established. Therefore, correct interpretation of imaging results should be based on neurological clinical signs to develop strategies to ensure that GSD patients have a longer and better-quality life^10,19,44–47,49^. Clinical examinations help clinicians interpret the radiological findings, understand their implications, and provide recommendations^5,48^. GSD neurological manifestations are associated with CNS^10,34,44,51–53^ and spinal nerve root and peripheral nerve involvement^26,27,44^; however, these manifestations may also be the result of concomitant diseases and can be detected only by clinical examination^4,6,43^.

SCI occurred more frequently than brain injury in this GSD study population, in accordance with the findings of other authors^6^.

Cervical SCIs were the most frequently found, as described by previous studies^2,3,5,21,54^. Among the GSDs, achondroplasia is most commonly associated with SCI and has been found in approximately 2 to 5% of patients^21,51,54^; however, other authors found a higher frequency of SCI in achondroplasia (28 to 33%)^5,44^. We also observed that patients with radiological spine abnormalities were more likely to have CNS injury, which is consistent with the findings of previous reports^2–5,10,19,21,44,52,53^.

CCJ abnormalities were common in our study population and were related to cervical SCIs, as reported by several authors^2,3,5,21^. BII in osteogenesis imperfecta and other GSDs^2,7,10,15,22^, a narrowed FM in achondroplasia^5,20,53,54^ and os odontoideum in bone dysplasias with prominent spinal involvement^24,25^ are potential causes of CNS injury. Imaging exams have shown that narrowed FM and spinal canal stenosis are frequent in GSD patients and may be associated with some degree of spinal cord compression without causing clinical manifestations, hydrocephalus and/or SCI^5,21,46^. In this study, cervical SCI associated with os odontoideum occurred in spondyloepiphyseal, spondylometaphyseal and metatropic dysplasia patients, and similar findings have been reported by other authors. Os odontoideum in GSD patients is associated with instability due to ligamentous laxity, bone tissue disorganization and delayed ossification of CCJ components^24,25^.

In the only patient with thoracic SCI in our study, there was a fixed kyphosis deformity and spinal cord compression and damage, which have also been reported in other studies^26,27,34^. Thoracic SCI may be related to thoracolumbar kyphosis in more than 10% of cases^26^. Thoracic intervertebral osteochondrosis and platyspondyly are also associated with CNS injury. Platyspondyly may lead to spinal canal narrowing and has been observed in some patients with GSDs if there is spondylodysplasia^2,3,15,17,27,55^.

Lumbar spinal canal stenosis may trigger spinal nerve root compression and neurological symptoms in the absence of SCI^44^. The frequency of neural compression in patients with different GSD types has been reported to be lower than the rates noted in achondroplasia patients^51,54^.

Hydrosyringomyelia has been detected by imaging in patients with different GSD types, without clinical manifestations^10^, and more frequently occurs in achondroplasia patients (12%)^21^.

Skull fractures caused encephalomalacia in osteogenesis imperfecta, and leukoencephalopathies in lysosomal storage diseases with skeletal involvement were causes of brain injuries in GSD patients, and these findings are consistent with those reported in a previous review^10^. Brain malformations and arachnoid cysts detected by imaging without clinical manifestations were abnormalities observed in GSD patients unrelated to a specific GSD^10^.

The presence of low back pain, ANPMD and os odontoideum was independently associated with the presence of CNS injury. The cause of neurological injury in GSD patients is multifactorial, according to previous studies^2,3,10^. CNS injury is more frequently associated with an abnormal configuration of the spine^2,10,15,17,18,21–25,52^, brain malformations^10,11^, leukoencephalopathies^2,10,12,13,55^, and spinal canal stenosis^2,10,15,17,18,20–25^. It is important to highlight that surgical trauma^44,56^, as well as the coexistence of other diseases, can also cause central and peripheral neurological damage in this population^44^.

ANPMD was observed in most of the GSD groups that were included in the study. Abnormal mineralization, lysosomal storage disease with skeletal involvement, osteogenesis imperfecta and decreased bone density, and chondrodysplasia *FGFR3* are the GSD types that may coexist with ANPMD and are frequently associated with CNS injury^10,21,55^.

A disproportionate short stature is a common characteristic of GSD patients, especially in achondroplasia, and our results showed a particularly high frequency of patients who were short and had a smaller wingspan among those who presented with CNS injury; however, these associations did not remain in the multivariable analysis.

Muscle weakness, the use of mobility aids and sensitivity abnormalities were not included in the uni- and multivariate analyses because when these manifestations were caused by neurological damage, they were included in the definition of the event of interest. Therefore, such analyses would result in incorporation biases. In this context, it is important to note that GSD can cause joint impairment and peripheral nerve injury, thereby affecting the neurological examination findings^42^.

The study's limitations are as follows: the sample selection in a rehabilitation center providing care to patients with orthopedic and skeletal deformities may have increased the relative frequency of CNS injury in the population studied; the exclusion of children under four years of age, since they require sedation for performing MRI and the anesthetic procedure is associated with risks incompatible with ethical aspects of the study; lack of socioeconomic data that would provide information on the causal relationships of individual characteristics; the small number of patients with each type of GSD, limiting the comparison of the CNS injury probability between different GSD types; the low frequency of spinal cord and brain injuries, which did not allow the study of specific etiopathogenic factors related to CNS injury; exclusion of patients with a severe and/or unstable clinical status, considering that the reference center for rehabilitation takes care of patients with stable clinical conditions; transversal characteristic of the study, which did not enable the evaluation of changes over time; the fact that a validated NPMD assessment tool was not used, although a developmental screening scale widely used for assessment was applied during the longitudinal follow-up at the institution; and transversal assessment of developmental abnormalities performed at the time of study.

## Methods

### Patient selection

This is an observational, cross-sectional, descriptive and analytical study that prospectively included patients who attended the rehabilitation center between January 2001 and December 2016. The project protocol was approved by the institution’s ethics committee (CAAE 49433215.5.0000.0022). All participants in the study signed informed consent forms. The study complied with the Declaration of Helsinki.

The GSDs studied were skeletal dysplasias (hereditary rickets, osteogenesis imperfecta, osteopetrosis, achondroplasia, metaphyseal dysplasias, diastrophic dysplasia, multiple epiphyseal dysplasias, spondyloepiphyseal dysplasias, spondylometaphyseal dysplasias, spondyloepimetaphyseal dysplasias and Schwartz-Jampel, for example), dysostoses (e.g., brachydactyly, cleidocranial dysplasia, spondylocostal dysostosis) and osteolyses^1^. The initial population consisted of 456 patients with congenital bone changes and orthopedic deformities following a review of medical records and confirmation of the presence of a GSD. After the patients and/or their legal guardians received information on the study and signed the informed consent form, they were invited to visit the institution and participate in the study. The inclusion criteria were GSD patients who were four or more years old. Patients who refused to participate in the study (164 patients) and those who were unable to undergo all the examinations (five patients) were not included. The exclusion criteria were a diagnostic coding error in the medical records (185 patients), loss to follow-up or the inability to be contacted (33 patients), peripheral nerve injury without CNS involvement (15 patients), death (six patients) and severe cognitive impairment (five patients). The required sample size was estimated based on 456 patients with a definitive diagnosis of GSD, the proportion of patients with CNS injury was 24.7%, the permissible error was 0.05, and the 95% confidence level was obtained, which resulted in a sample size of 176 patients.

### Clinical and radiological evaluations

Based on clinical and radiological reassessments of the medical records, the patients were grouped using the International Society of Skeletal Dysplasia (ISSD)^50^ guidelines. The diagnosis of *FGFR3* chondrodysplasias was confirmed by genetic testing. Clinical, radiologic and laboratory diagnosis of hypophosphemic rickets was confirmed by serum levels of calcium, phosphate, alkaline phosphatase, 1,25-dihydroxyvitamin D, parathyroid hormone and creatinine, as well as by urinary phosphate and creatinine levels. The diagnosis of lysosomal storage diseases with skeletal involvement required enzymatic tests in patients with radiographic features of dysostosis multiplex. Thus, the following tests were performed: urinary excretion of mucopolysaccharids, oligosaccharides and sialic acid and activity of leucocytes, beta-galactosidase and beta-hexosaminidas.

All participants underwent medical consultations, imaging examinations and laboratory tests on the same day. The results of spine DR, dynamic CCJ CT and brain and spinal cord MRI (1.5T) performed on all participants were analyzed. Clinical and radiological evaluations of conditions including neuropsychomotor development (NPMD), were independently performed for each patient by the same multidisciplinary team. NPMD was assessed longitudinally by medical records data^57^ and transversely by the team on the day the patients were recruited. The educational level of the participants was also considered. The anthropometric data were measured by two physicians, including weight, stature, lower segment length, upper segment length, trunk length, wingspan, occipitofrontal circumference (OFC), and body mass index (BMI). Cases with spinal cord injuries diagnosed clinically or by imaging examinations were classified according to the 2019 version of the International Standards for Neurological Classification of Spinal Cord Injuries (ISNCSCI)^42^. Imaging exams were evaluated by two radiologists with 23 years of experience at a remote workstation, without knowledge of clinical data. Blinded and systematic CCJ evaluation using craniometrical parameters was performed with the three imaging modalities (DR, CT and MRI) by two radiologists at three different time points. The reference values of the CCJ parameters were selected based on a literature review.

The patients were divided into two groups, namely, those with and without CNS injury. Patients with CNS injury were characterized by abnormal imaging and/or neurological examination. If a focal or diffuse T2-hyperintense signal or a brain malformation was found, there was brain injury. If imaging showed hydrosyringomyelia and/or myelomalacia, SCI was considered. Additionally, there were patients with spinal cord injury without radiographic abnormalities (SCIWORA). Two patients with neurological disorders caused by vitamin B12 deficiency and schistosomal myeloradiculopathy were not considered to have GSD and CNS injury.

### Data and statistical analysis

The data were analyzed using Hornik, “The R FAQ” (https://CRAN.R-project.org/doc/FAQ/R-FAQ.htmL)58 statistical programming software, which is open source. The variables included in the analysis comprised the clinical, laboratory and imaging findings. The nominal variables are expressed as numbers and percentages. The continuous variables are presented as medians and IQRs, as the Shapiro–Wilks test revealed that most of these variables were nonnormally distributed. The chi-square (X^2^) test and Fisher’s exact test were used to compare the categorical variables between the groups with and without CNS injury, and the Mann–Whitney–Wilcoxon test was used to compare the medians. Odds ratios (ORs) with 95% confidence intervals (CIs) were calculated for each variable. Variables corresponding to a *p* value of less than 0.20 in the univariate analysis were included in a multivariate model. The associations between the clinical and radiological variables and the event of interest (CNS injury) were assessed using logistic regression following the backward technique. The Hosmer–Lemeshow test was used to verify the adequacy of the final model.

## Supplementary Information

Supplementary Informations.

## Data Availability

The data are available at Rede SARAH (400564@sarah.br).
