# Supplementary material for: Identification of clinical and radiographic predictors of central nervous system injury in genetic skeletal disorders
Source: Sci Rep. 2021 May 31;11:11402. doi: 10.1038/s41598-021-87058-5 (PMC8166875; doi:10.1038/s41598-021-87058-5)
Supplement: Supplementary file 1 — Supplementary Informations. [file 41598_2021_87058_MOESM1_ESM.docx]

**Supplementary material**

**Supplementary Table S1.** Univariable analysis of predictors of CNS injury in genetic skeletal disorder patients

| Predictors | p* | OR (95% CI) |
| --- | --- | --- |
| Low back pain | 0.003 | 2.3 (1.3-4.0) |
| Lumbar platyspondyly | 0.003 | 2.9 (1.5-5.9) |
| Os odontoideum | 0.004 | 3.9 (1.5-9.8) |
| Cervical platyspondyly | 0.006 | 2.8 (1.3-5.8) |
| Thoracic platyspondyly | 0.006 | 2.6 (1.3-5.2) |
| Radiological spine abnormalities | 0.006 | 2.2 (1.2-3.7) |
| Abnormal neuropsychomotor development | 0.008 | 2.8 (1.3-6.2) |
| Platyspondyly | 0.016 | 2.3 (1.2-4.5) |
| Basilar impression/invagination | 0.018 | 2.4 (1.2-5.0) |
| Thoracic osteochondrosis intervertebral | 0.021 | 2.0 (1.1-3.5) |
| Epiphyseal abnormalities | 0.034 | 1.9 (1.1-3.5) |
| Narrowed foramen magnum | 0.036 | 2.2 (1.1-4.7) |
| Metaphyseal abnormalities | 0.044 | 0.6 (0.3-1.0) |
| Stature | 0.045 | 1.0 (1.0-1.0) |
| Wingspan | 0.049 | 1.0 (1,0-1.0) |
| Radiological skull abnormalities | 0.064 | 2.0 (1.0-4.2) |
| Lower limb discrepancy | 0.072 | 1.7 (1.0-2.8) |
| Thoracolumbar kyphosis | 0.079 | 1.9 (0.9-3.8) |
| Body mass index | 0.083 | 1.0 (0.9-1.0) |
| Occipitofrontal circumference | 0.086 | 0.9 (0.8-1.0) |
| Upper segment length | 0.091 | 1.0 (1.0-1.0) |
| Scoliosis | 0.144 | 0.7 (0.4-1.1) |
| Cervical osteochondrosis intervertebral | 0.149 | 1.5 (0.9-2.6) |
| Decreased bone density | 0.160 | 0.6 (0.3-1.1) |
| Osteochondrosis intervertebral | 0.209 | 1.4 (0.8-2.5) |
| Atlanto-odontoid instability | 0.290 | 1.8 (0.6-5.0) |
| Spinal canal stenosis at C2 | 0.292 | 1.5 (0.7-3.0) |
| Thoracic spinal canal stenosis | 0.316 | 1.4 (0.7-2.7) |
| Diaphyseal abnormalities | 0.317 | 1.4 (0.7-2.9) |
| Ligament laxity | 0.347 | 1.4 (0.7-2.8) |
| Joint dislocation | 0.376 | 1.6 (0.6-4.5) |
| Lumbar osteochondrosis intervertebral | 0.389 | 1.3 (0.7-2.4) |
| Cervical spinal canal stenosis | 0.389 | 1.3 (0.7-2.3) |
| Joint deformity | 0.426 | 1.3 (0.7-2.5) |
| Disorganized bone development | 0.461 | 0.8 (0.4-1.6) |
| Spinal vertebrae compression | 0.465 | 0.8 (0.4-1.6) |
| Trunk length | 0.482 | 1.0 (1,0-1.0) |
| Thoracic vertebrae compression | 0.517 | 0.8 (0.4-1.7) |
| Spine canal stenosis | 0.520 | 1.2 (0.7-2.1) |
| Lower segment length | 0.530 | 1.0 (1.0-1.0) |
| Cervical spinal cord compression | 0.531 | 1.2 (0.7-2.3) |
| Weight | 0.544 | 1.0 (1.0-1.0) |
| Atlanto-occipital instability | 0.617 | 1.4 (0.3-5.9) |
| Increased bone density | 0.629 | 0.6 (0.4-4.2) |
| Terminal bone at C2 | 0.630 | 1.5 (0.1-1.1) |
| Basio-occipital hypoplasia | 0.687 | 0.9 (0.5-1.6) |
| Lumbar vertebrae compression | 0.707 | 0.8 (0.3-2.3) |
| Headache | 0.767 | 0.9 (0.5-1.6) |
| Age | 0.910 | 1.0 (1.0-1.0) |
| Lumbar spinal canal stenosis | 0.921 | 1.0 (0.5-2.0) |
| Gender | 0.976 | 1.0 (0.6-1.7) |
| Cervical vertebrae compression | 0.984 | 0.0 (0.0-4.6) |
| Platybasia | 1.000 | 0.8 (0.1-7.8) |

CNS: central nervous system; * X^2^ test or Fisher’s exact test. OR: odds ratio; CI: confidence interval.

**Supplementary Table S2.** Multivariable analysis predictors of CNS injury in genetic skeletal disorder patients before and after adjustments

| Predictors/adjustments | p* | p1st | p2nd | p3rd | p4th | p5th | p6th |
| --- | --- | --- | --- | --- | --- | --- | --- |
| Abnormal neuropsychomotor development | 0.023 | 0.020 | 0.030 | 0.021 | 0.016 | 0.020 | 0.012 |
| Low back pain | 0.091 | 0.091 | 0.077 | 0.052 | 0.057 | 0.041 | 0.021 |
| Os odontoideum | 0.189 | 0.179 | 0.151 | 0.113 | 0.078 | 0.053 | 0.022 |
| Thoracic osteochondrosis | 0.355 | 0.348 | 0.332 | 0.294 | 0.319 | 0.946 | - |
| Radiological spine abnormalities | 0.370 | 0.371 | 0.301 | 0.303 | 0.190 | - | - |
| Narrowed foramen magnum | 0.409 | 0.400 | 0.417 | 0.326 | - | - | - |
| Basilar impression/invagination | 0.480 | 0.481 | 0.475 | - | - | - | - |
| Platyspondyly | 0.811 | 0.816 | - | - | - | - | - |
| Epiphyseal abnormalities | 0.957 | - | - | - | - | - | - |

CNS: central nervous system; R^2^ = 10.9%; Hosmer-Lemeshow test = 0,995; * X^2^ test or Fisher’s exact test.
